# A dataset for studying the vocabulary of picturebooks: A supplement to the children’s picturebook lexicon (CPB-Lex)

**DOI:** 10.1016/j.dib.2026.113200

**Published:** 2026-08-27

**Authors:** Clarence Green, Kathleen Keogh

**Affiliations:** a Faculty of Education, University of Hong Kong, Hong Kong, China; b Institute of Innovation Science and Sustainability, Federation University, Australia

**Keywords:** Vocabulary, Literacy, Reading, Picture books, Modelling, Psycholinguistics

## Abstract

This data-in-brief article shares a large-scale lexical resource derived from children’s picturebook read‑aloud videos publicly available online, supplementing the related Children’s Picturebook Lexicon (CPB‑Lex) by substantially increasing corpus size, and providing richer metadata such as genre information. Using automatically generated English closed captions as a proxy for picturebook text, we collected, filtered, and processed 5874 unique read‑aloud transcripts, yielding a dataset of approximately 3.49 million word tokens and 44,617 word types. Each transcript was tokenized and aggregated into document–term matrices (DTMs), one representing the full corpus, and additional DTMs for narrative and informational subsets defined via genre labels assigned by a large language model. From these lexical resources, word, lemma and text frequencies are derived and shared. Additional metadata include video titles, source channels, and view counts (where available), supporting analyses of the different types of picturebook language input patterns for educational, computation and cognitive science research. The dataset enables researchers to estimate children’s exposure to specific words and lexical distributions in early shared reading, to select stimuli for experimental work (e.g., lexical decision or naming tasks), and to model aspects of vocabulary growth and reading development. The data is openly available on Mendeley Data, the Open Science Framework and a webpage platform for data sharing: https://cgg-projects.github.io/CPB_LEX/.

Specifications Table

**Table:** 

Subject	Social Sciences
Specific subject area	Educational research; reading research; psycholinguistics; language exposure and vocabulary; computational / corpus‑based modelling
Type of data	Document–term matrices (CSV); word frequency lexica (Excel); metadata tables (Excel); processing code and scripts (Python)
Data collection	Lexical statistics were derived from automatically generated English closed captions of publicly available picturebook read‑aloud videos on YouTube. Only subtitle text and metadata were downloaded; no audio or video files were collected. Captions were then cleaned, tokenized, and aggregated into document–term matrices and frequency lexica using Python scripts (provided in the repository).
Data source location	The underlying source material consists of publicly available YouTube read‑aloud videos, hosted on YouTube’s global platform (no single geographical location; channels are predominantly U.S.-based but international in scope). The original ASR transcripts and videos are not stored long term or redistributed. Only derived datasets (DTMs, lexical, and metadata) are stored and shared via the Open Science Framework.
Data accessibility	Repository name: Mendeley Data and the Open Science Framework (OSF) Data identification number: [Reserved DOI: 10.17632/9pgmnfkf56.1] Direct URLs to data: Mendeley Data: DOI: 10.17632/9pgmnfkf56.1 Open Science Framework: https://osf.io/53e9y/overview?view_only=c4aff93894c142ecbefe1f225373672c All data and code required to reproduce the derived datasets are available in Mendeley Data and the OSF project. No login is required We have developed a platform webpage to share the data: https://cgg-projects.github.io/CPB_LEX/
Related research article	Green, C., Keogh, K., Sun, H., & O’Brien, B. (2024). The Children’s Picture Books Lexicon (CPB-LEX): A large-scale lexical database from children’s picture books. *Behavior Research Methods*, 56(5), 4504–4521.

## 1 Value of the Data

• This dataset provides a large-scale representation of the vocabulary in children's picture books as encountered in early print and read-aloud environments (using an online social media proxy). Picture books have been linked to various positive language and literacy outcomes, yet large-scale characterizations of their lexical properties remain limited compared to other text types.
• The dataset supplements the published project Green et al. (2024) [1], namely the Children’s Picturebook Lexicon (CPB-Lex), a frequency-lexicon of words derived from automatically generated captions of social media picturebook read-alouds. This new dataset builds on that idea but is much larger, includes data from instructional and narrative picture books and additional useful metadata for researchers.
• The core datafile is shared as a document-term matrix that can be reused to derive word, text and corpus level distributional statistics for modelling print exposure, age of acquisition/exposure estimates, and other predictive models in reading research. Researchers can combine the data with existing resources to estimate lexical properties children encounter in shared reading.
• This dataset, along with CPB-Lex, provides authentic vocabulary that may be useful in cognitive modelling and computational simulation studies, including the development of artificial ‘child’ agents that can be used to investigate children’s language acquisition based on exposure to picture books. AI-based agent simulation models of children could be a powerful and customisable platform to explore the potential impact of behavioural and social factors on language development and literacy, complementing human studies.
• The resource supports selection of experimental stimuli. For example, words for lexical decision tasks, naming experiments, or updating tests that currently rely on smaller or outdated sources.
• In addition of genre labels (narrative vs. informational), in addition to titles, source channels, and view counts, allows users to isolate subsets of texts or to study popularity-based sampling in read‑aloud contexts. This makes it possible to investigate how genre and popularity relate to children’s lexical input.
• The dataset fills a gap in existing databases that focus on early print environments. Whereas prior corpora have concentrated on chapter books, independent reading materials, or K–12 textbooks, this resource specifically targets picturebooks read to young children and thus complements existing materials for modelling children’s early literacy experiences.

## 2 Background

Researchers are increasingly interested in the linguistic properties of children’s early language environments and print exposure to inform theories of language acquisition, reading development, and instruction [[1], [2], [3], [4], [5]]. Large-scale resources describing picturebook language, the primary form of early book exposure via shared reading [6], have been relatively scarce, partly due to the costs of scanning and transcribing many short texts [7].

The Children’s Picturebook Lexicon (CPB-Lex) [1] addressed this gap by extracting lexical statistics from automatically generated captions on publicly available social media YouTube channels in which adults read picturebooks to a virtual audience of children, yielding a corpus of approximately 1.1 million word-tokens across 2146 unique readings and about 25,585 word types.

The present data article documents a supplementary corpus that substantially expands CPB‑Lex in scale and metadata for a lexical database from 5874 picturebooks- over 2.5 times larger. The new dataset is based on a larger collection of read‑aloud videos, incorporates genre classifications (narrative vs. informational), and includes additional text-level metadata such as view counts. Sharing this dataset offers a broadened resource for studying the vocabulary of children’s picturebooks and for modelling early print input.

## 3 Data Description

The data are hosted at the Mendeley Data repository (DOI: 10.17,632/9pgmnfkf56.1), as well as the Open Science Framework (OSF) under creative commons licences: https://osf.io/53e9y/overview?view_only=c4aff93894c142ecbefe1f225373672c. Both the repositories are organized into two main components: (a) data files and (b) code used to construct the dataset. The core of the dataset is the document term matrices (DTM) that represent the lexical statistics of the proxy for picturebooks. In each DTM, the word (lowercase) is provided in the first column, and the columns each represent one document (i.e. one picturebook read-aloud transcript from automatically generated closed captions). Table 1 provides an overview of the main data files.

**Table 1:** Structure of the dataset.

Repository file / folder	File type	Description
dtm_all.csv	Document–term matrix (CSV)	Document–term matrix for the full corpus. Each row corresponds to a unique word type (lowercase). Each column from text_1 onward corresponds to a single picturebook read-aloud transcript. Each cell contains the raw frequency of that word in that transcript.
dtm_narrative.csv	Document–term matrix (CSV)	As above, but restricted to transcripts labelled as narrative picturebooks. Only narrative documents are included as columns.
dtm_informational.csv	Document–term matrix (CSV)	As above, but restricted to transcripts labelled as informational (non-fiction / expository) picturebooks. Only informational documents are included as columns.
lexicon_frequencies.xlsx	Lexicon (Excel spreadsheet)	Word-level frequency lexicon. Contains nine columns: (1) wordform, (2) lemma, (3) frequency in the full corpus, (4–5) wordform and lemma frequencies in the narrative subset, and (6–7) wordform and lemma frequencies in the informational subset, plus additional summary columns (e.g., document frequencies) where applicable.
metadata_view_counts.xlsx	Metadata (Excel spreadsheet)	File-level metadata for the transcripts. Rows represent individual transcripts (picturebook read-alouds). Columns include: transcript filename / ID, YouTube video title, source channel name, and view count (where available). Entries for which no view count could be located are coded as missing.
metadata_genre_labels.xlsx	Metadata (Excel spreadsheet)	Genre labels for each transcript. Contains transcript filename / ID, video title, and a categorical genre label (NARRATIVE or INFORMATIONAL) assigned by a large language model (GPT-4.1).
source_channels.txt	Plain text	List of YouTube channels from which read-aloud transcripts were collected. Each line contains a channel name and/or URL. This file documents the source space of the corpus.
titles_list.txt	Plain text	Complete list of social-media video titles corresponding to the transcripts underlying the DTMs. Each line contains the title as displayed on YouTube; in many cases this includes the picturebook title and additional channel-specific information.
code/	Folder (Python scripts, etc.)	Code used to construct and process the dataset. This includes scripts for (a) downloading closed captions, (b) filtering and cleaning transcripts, (c) tokenization and lemmatization, (d) building DTMs, and (e) generating the frequency lexicon and metadata tables. Configuration files (e.g., requirements) are provided to facilitate reproducibility and extension.

All DTMs are sparse (most word–document combinations are zero), but stored as dense CSV files with empty or zero cells corresponding to absences. Word order within documents is not preserved in these matrices. The repository does not redistribute full running texts of the picturebooks as a corpus. Only derived data products are shared: DTMs, word-level lexical statistics, and file-level metadata. The automatically generated subtitle transcripts themselves are not stored or shared long term in the repository. The accompanying code allows users to (a) reconstruct the pipeline on newly downloaded subtitles, (b) extend the corpus with additional read-alouds, or (c) regenerate the DTMs and lexical data under modified pre-processing choices.

The texts considered narrative picturebooks were primarily fictional stories, rather than informational texts focus on real-world facts, historical events, animals, and related content [[8], [9], [10]]. Examples of narrative picturebooks in the corpus include titles such as *Pete the Cat, The Very Hungry Caterpillar*, and *Paw Patrol* read‑aloud to a virtual online audience. Examples of informational picturebooks include titles such as *Meet the Animals – Zebra, Flutter, Butterfly! (National Geographic)*, and *Bears – a nonfiction book read aloud* etc. In practice, the boundary between narrative and informational texts is sometimes fuzzy, for instance in books that personify animals or natural phenomena (e.g., “a day in the life of a bee”) or titles such as *If Sharks Disappeared*, which are fictional or entertainment‑oriented but also aim to convey to children some scientific or environmental information.

**Fig. 1:**
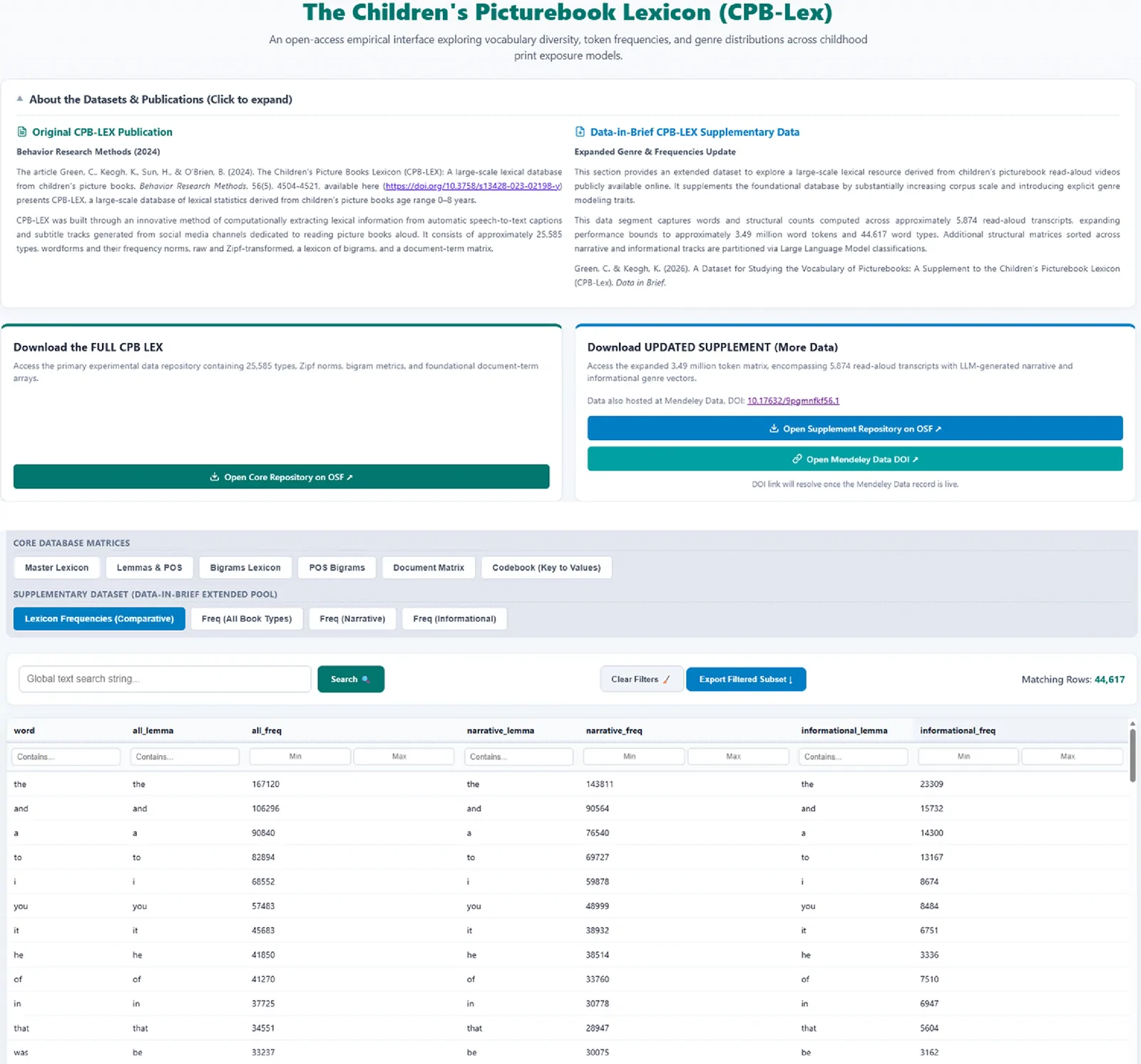
The data-sharing portal interface.

In addition, we have developed an open-access, interactive data-sharing platform hosted as a companion web portal at https://cgg-projects.github.io/CPB_LEX/. This digital interface is engineered to facilitate data use. It combines both the current data and original CPB-LEX, as illustrated in Fig. 1.

The portal utilizes a lazy-loading architecture that allows researchers to quickly switch between different tabs containing the core database matrices (e.g., Master Lexicon, Lemmas & POS, Bigrams Lexicon, POS Bigrams, and the Document-Term Matrix) as well as the comparative supplementary datasets partitioned by genre. Within the web interface, users can leverage an integrated search and discovery toolkit featuring text string queries alongside column-specific metadata filters, including custom minimum and maximum numeric parameter boundaries for frequency counts. To accommodate researchers and use cases, the platform includes a customized compilation extraction feature, enabling users to isolate a filtered subset on screen and export it directly as a standalone CSV file.

## 4 Experimental Design, Materials and Methods

In this section, the research design, materials and methods used in development of the dataset are described. We first identified YouTube channels that systematically host picturebook read‑aloud videos, using search terms such as “read aloud”, “picturebook”, and related phrases in video titles and channel descriptions. The focus was on English‑language content targeting young children. This process yielded 59 target channels, listed with links in the OSF repository. For comparison, the original CPB‑Lex corpus [1] was based on 33 channels, so the present dataset draws on a substantially larger pool of sources.

Closed captions and subtitles were then collected from the identified channels using the open‑source tool yt‑dlp (https://github.com/yt-dlp/yt-dlp), a comprehensive Python package widely used in research [11]. For each video, English subtitles and closed captions were requested. No video or audio files were downloaded; only subtitle text and essential metadata (e.g., title, channel, duration, video ID) were retained for processing. All data collection and pre-processing code is provided in the OSF repository. The method follows the procedure used for CPB‑Lex [1], which leveraged the emerging genre of picturebook read‑alouds on social media and automatic speech recognition (ASR) to construct a large corpus of picturebook vocabulary. Previous work has reported high accuracy for YouTube’s ASR [1,10], though accuracy can be affected by pronunciation, background noise, accents, and dialectal variation.

The initial data collection method adopted produced a pool of 11,524 subtitle transcripts. Subsequently, several filters were applied to that data. First, lengthy recordings (typically exceeding 20 min) were removed as these typically included multiple books or extended non-book content. Second, on the remaining dataset of transcripts for read-alouds under 20 min, video titles were inspected and materials that were clearly not picturebook readings (e.g., general channel introductions, non-story videos) were excluded. To reduce presenter speech (e.g., greetings, instructions, channel sign-offs) and better isolate book language, the first 20 s and last 10 s of each transcript was trimmed. The code has been placed in the OSF folders for other researchers. These steps parallel the cleaning procedures described in [1] although in that study transcripts were manually inspected and trimmed by research assistants.

Duplicate readings of the same book were then identified and removed. This was achieved by program code implemented to estimate lexical overlap between transcripts (e.g., via similarity over word distributions) and flag highly similar files as potential duplicates; these identified duplicates were resolved by retaining a single representative transcript per title. This process was supplemented by manual inspection where necessary. After filtering and deduplication, 5722 transcripts remained. In addition, we incorporated 152 informational picturebook transcripts from [10], which were manually downloaded closed captions from targeted searches for informational picturebooks. The final corpus thus comprises 5874 unique picturebook readings.

Given interest in how informational and narrative texts shape children’s language environments, the new dataset includes genre labels for each text. Each transcript was assigned an estimated genre label, either -narrative (story‑like, with characters and events) or informational (non‑fiction/expository). Genre assignment combined two steps: (1) automatic classification, where titles were submitted to a large language model (GPT‑4.1) which was prompted to behave like an expert in children’s literature and constrained to output either “NARRATIVE” or “INFORMATIONAL”; and (2) curated additions, where the 152 informational picturebooks from [10] were explicitly assigned to the informational category manually. The model outputs were compared with ground truth for plausibility of the automated classifications generated by the LLM. The first author wrote code to create a stratified random sample of 100 LLM suggested genre attributions (50 NARRATIVE/50 INFORMATIONAL) with the output file shuffling the data and leaving out the classifications. The titles were then blindly tagged by the first author as NARRATIVE or INFORMATIONAL, without looking them up online. This was done to be consistent with the LLM prompt (though the LLM was also told it could draw on its knowledge-base of the text, so may have performed better than human annotation that did not search the title online). There was 91% agreement between the LLM and author 1 on suggested genres. Disagreements were interesting, reflecting borderline cases, such as a picturebook about the Year of the Snake in the Chinese Zodiac being classified by the LLM as narrative while the first author labelled it as informational - both are reasonable. In other cases of non-alignment, the LLM seemed more accurate, possibly because its training data knew these texts.

The LLM labels were used to partition the corpus and construct separate DTMs for narrative and informational texts. The code used to call the LLM and suggest if a text was more informational or more narrative in genre is provided in the OSF, as is the code used for stratified randomly sampling and the output of the blind spot checking.

Text‑level metadata were compiled for each transcript and are provided in the shared dataset. The available fields include the video title (as displayed on YouTube), source channel, genre label (narrative vs. informational), and view count (where available). View counts were not collected via code, given programming difficulty and instead, a research assistant manually retrieved view counts from the corresponding pages for the collected titles. Because some filenames included emojis or other characters that complicated the RAs direct URL matching, we are only able to provide view counts for 5229 picturebook read-alouds. We do not release individual linked corpus files, as appropriate for fair use/dealing, so the metadata is provided in spreadsheets. The main provision of this metadata is for researchers to better understand what went into the database, such as the popularity of the underlying picturebooks, their titles, as well as if they decide to add to reproduce it what title have already been captured in this dataset.

The final ∼5874 transcripts were then tokenized with Spacy (https://spacy.io/) in python and normalized via lowercasing) via code that also constructed document–term matrices (DTMs) for each corpus subset. In these matrices, each column corresponds to one unique picturebook reading, each row corresponds to a distinct word type, and each cell contains the raw frequency of that word in that text. Separate DTMs were created for the narrative and informational subsets, as well as an overall DTM combining both. Because the representation is bag‑of‑words, word order is not preserved and the original running text cannot be reconstructed from the DTMs.

The DTMs can be used to derive many lexical statistics at the type and corpus level, such as token frequencies and document frequencies. We provide core frequency lists as part of the data release that follow the structure of CPB‑Lex earlier resource [1]. Using Spacy, we generated a frequency list of word tokens and their lemmas in a file that contains an overall frequency-ranked wordlist. The present data article focuses on documenting the corpus and DTMs; users can use the provided matrices to compute additional measures such as dispersion indices [12], inverse document frequency (IDF) [13], or frequency‑based age‑of‑exposure proxies [14].

Table 2 summarizes the product of the above-described procedures in terms of key lexical statistics for the informational, narrative, and combined corpora. The data reported here can be derived from the dataset we have shared.

**Table 2:** Summary of lexical statistics for the shared dataset.

corpus	n_files	total_tokens	vocab_size (types)
informational	930	528,382	19,749
narrative	4944	2956,620	39,970
combined	5874	3485,002	44,617

The original CPB-Lex corpus[1] comprised approximately 1120,778 tokens across 2146 picturebook readings and about 25,585 word types. The present supplementary dataset thus more than triples the number of tokens and substantially increases both the number of texts and the vocabulary size relative to CPB‑Lex, while also providing genre labels and additional metadata.

## Limitations

The corpus is a proxy for picturebook language and inevitably contains both signal and noise. Because captions were taken from full read-aloud videos, it was not possible to completely isolate book text from reader speech (e.g., greetings, instructions, commentary). Time-based trimming of openings and closings reduces but does not eliminate this contamination.

All language is derived from YouTube’s automatic speech recognition, which is generally accurate but can mis-transcribe words, particularly under variation in accent, recording quality, or background noise. These errors introduce additional noise into lexical counts.

Genre labels (narrative vs. informational) were assigned using a general-purpose large language model (GPT-4.1) applied to titles and minimal context. The model was not fine-tuned or separately validated for children’s genre classification, so some misclassification is likely, especially for hybrid texts that mix narrative and expository features.

The dataset is further limited by its sampling frame: channels are predominantly U.S.-based, and view counts are missing for a subset of titles. Finally, unlike the original CPB-Lex, no psychometric validation (e.g., against behavioural benchmarks) has yet been conducted for this supplementary dataset; it is provided primarily as a larger, complementary lexical resource.

## Ethics Statement

This study did not involve human participants nor require ethics approval. All data were obtained from publicly available social media content and platform data use and redistribution policies have been followed.

## CRediT Author Statement

**Clarence Green:** Database Conceptualization, Methodology, Coding; Writing; **Kathleen Keogh:** Database Conceptualisation; original coding; Reviewing and Editing.

## Declaration of Generative AI and AI-Assisted Technologies in the Manuscript Preparation Process

During the preparation of this work GPT 4.1. was used in order to assist with writing code and language editing. After using this tool/service, the authors reviewed and edited the content as needed and take full responsibility for the content of the published article.

## Data Availability

Mendeley DataA Supplement to the Children’s Picturebook Lexicon (CPB-Lex) (Original data). Open Science FrameworkCPB-LEX (Original data).
